# Laser ablation process of CsPbBr_3_ heterostructures for light-emitting diode applications

**DOI:** 10.1080/14686996.2025.2554045

**Published:** 2025-09-09

**Authors:** Ryunosuke Kumagai, Ren Koguchi, Takuro Dazai, Toshihiro Sato, Hideomi Koinuma, Ryuzi Katoh, Ryota Takahashi

**Affiliations:** a College of Engineering, Department of Electrical and Electronic Engineering, Nihon University, Fukushima, Japan; b Vacuum Products, Tokyo, Japan; c Smart Combinatorial Technology, Tokyo, Japan; d College of Engineering, Department of Chemical Biology and Applied Chemistry, Nihon University, Fukushima, Japan

**Keywords:** Halide perovskite, pulsed laser deposition, time resolved microwave conductivity, time resolved photoluminescence, light emitting diode

## Abstract

We investigated a vacuum thin-film process using laser ablation to fabricate heterostructures of halide perovskite CsPbBr_3_ for light-emitting diode (LED) applications. A CsPbBr_3_ single crystal synthesized via inverse temperature crystallization was used as the target material for pulsed laser deposition. CsPbBr_3_ films were deposited at 150°C, 200°C and 250°C. Structural and optical analysis has revealed that the optimum temperature is 200°C, which display the highest crystallinity and photoluminescence emission efficiency. Time-resolved microwave photoconductivity characterization revealed that the CsPbBr_3_ film exhibited a high effective mobility of 2.47 cm^2^/Vs and long photocarrier lifetime of 16.5 μs. The lifetime is comparable to that of bulk CsPbBr_3_ single crystals. This indicates that the polycrystalline CsPbBr_3_ film had a low density of defect structures that promote nonradiative recombination. Furthermore, we applied this process to fabricate a LED device using halide perovskite heterostructures. This resulted in a strong green electroluminescence emission. The laser ablation process using ultraviolet and infrared light is suitable for forming heterostructures with an electron transportation layer of oxide Mg_0.3_Zn_0.7_O film and a hole transportation layer of an organic α-NPD film. The film synthesis process is likely to be effective for evaluating heterointerfaces of various materials displaying remarkable crystallinity without exposure to air.

## IMPACT STATEMENT

A thin film process was developed to significantly enhance the photoconductivity of the effective mobility and photocarrier lifetime in halide perovskite films, and employed for application to the LED devices.

## GRAPHICAL ABSTRACT

**Figure:**
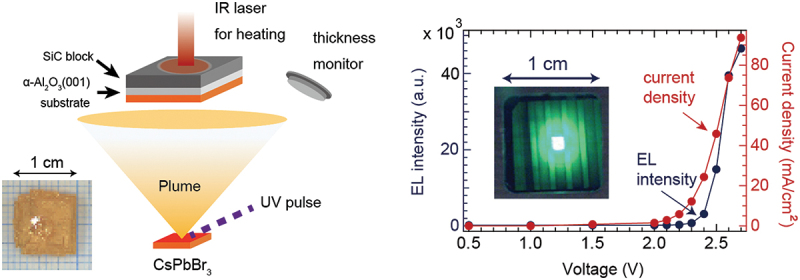

## 1. Introduction

Halide perovskite materials exhibit defect tolerance. This is an important property for application in photovoltaic devices [1–4]. Even if defect structures are formed in the crystal to a certain extent, the functional properties of the halide perovskite films and devices do not deteriorate significantly. This is attributed to the characteristic electronic structure of the halide perovskite crystals [5,6]. The valence and conduction bands of halide perovskites are composed of antibonding electronic structures. Therefore, the defect level in the crystal is unlikely to be deep, and carrier recombination is unlikely to occur. Therefore, the negative factors that deteriorate the energy conversion between light and electricity can be minimized. Consequently, halide perovskite films with remarkable optical properties have been synthesized with good reproducibility via the solution-coating process, wherein it is difficult to obtain high mobility and long photocarrier life in standard semiconductors such as GaAs and GaN. Therefore, halide perovskite films have attracted considerable attention for applications in solar cells [7–9], light-emitting diodes (LED) [10–12], and laser crystals [13–15].

Improving the crystallinity of semiconductor films is key to improving the functional properties and, thereby, the enhancement of device performance. This tendency is particularly evident for standard semiconductors. Improving the crystallinity reduces the likelihood of nonradiative recombination and lengthens the nonradiative recombination time. Consequently, the photocarrier lifetime increases, thus yielding improved photoluminescence (PL) properties [16,17]. Therefore, the photocarrier lifetime is an important parameter that strongly influences the device properties of film crystals such as solar cells, LEDs, and photocatalytic materials. The typical examples include ZnO [16] and GaN [17] thin films that are widely used as laser materials and LEDs. Improving the crystallinity of these thin films increases the nonradiative recombination time. This, in turn, yields improved luminescence properties and device performance. Similar improvements in photoelectric conversion efficiency have also been demonstrated in water-splitting photoelectrodes [18,19] and in photoconductivity [20] using semiconducting SrTiO_3_ films.

Recently, halide perovskites have been shown to be defect tolerant for cold carriers at the band edge. However, there is considerable debate as to whether these also exhibit defect tolerance for hot carriers [1]. If the formation of halide perovskite films with lower defect densities is feasible, as in commercial semiconductors, the potential of halide perovskite materials can be increased dramatically. This can result in improvements in the functional device properties of solar cells and LEDs based on halide perovskite materials. Dry vacuum processes such as pulsed laser deposition (PLD) [21–26], infrared laser (IR) molecular beam epitaxy (MBE) [27–30], and vacuum evaporation [13,31–35] have been developed for this purpose. Vacuum processes are likely to suppress the contamination by water and impurities, thereby producing halide perovskite films of higher purity and better crystallinity.

For the IR-MBE process [27–30], the halide powder is mixed with silicon powder (which absorbs IR light significantly) and placed in a vacuum. The silicon powder is heated by irradiation with an IR laser. The raw halide material evaporates under vacuum. This enables the gentle deposition of a halide perovskite film on the substrate similar to the MBE method. By modulating the frequency and power density of the IR laser, it is feasible to control the deposition rate using a proportional-integral-derivative (PID) controller. This enables the synthesis of materials at any speed and with atomic-level control of the film thickness. IR-MBE deposition has also been applied to the synthesis of representative halide perovskite films such as CH_3_NH_3_PbI_3_^28,29^, CsPbBr_3_^27,30^, and CsPbI_3_^30^. In particular, a time-resolved microwave photoconductivity (TRMC) measurement revealed that the photocarrier lifetime of CsPbBr_3_ thin films deposited by IR-MBE is 35 μs. This is over 100 times longer than that of thin films synthesized by the solution-coating process [27]. The photocarrier lifetime is a crucial parameter determined by the balance between radiative and nonradiative recombination. It can be increased by reducing the density of defect structures that cause nonradiative recombination. Therefore, the long photocarrier lifetime demonstrates the feasibility of synthesizing highly crystalline CsPbBr_3_ films via a dry vacuum process using an IR laser.

However, in IR-MBE deposition of CsPbBr_3_ films on α-Al_2_O_3_(0001) substrates, the CsPb_2_Br_5_ phase (which is deficient in CsBr) is mixed into the CsPbBr_3_ film. Contamination of the CsPb_2_Br_5_ phase deteriorates the optical properties such as light absorption, PL emission, and photoconductivity [27]. PLD using an ultraviolet (UV) laser is a potential method for preventing CsBr deficiencies. A pulsed UV laser with a pulse width of the order of nanoseconds is focused on the target material. Subsequently, the raw material is evaporated under vacuum to deposit a CsPbBr_3_ film [21–26]. Compared with the IR-MBE process, PLD is likely to prevent the precipitation of the impurity CsPb_2_Br_5_ phase by instantaneously evaporating the CsPbBr_3_ raw material at a high deposition rate. However, PLD requires solid target materials for laser ablation. The low density of the target material results in a low film crystallinity. However, it is generally difficult to synthesize a target halide perovskite material with a high sintered density ( >95%) for the PLD synthesis of oxide films.

In this study, we synthesized CsPbBr_3_ single crystals using the inverse temperature crystallization (ITC) method [36–39]and applied the single crystals as a target for PLD synthesis. Using CsPbBr_3_ single crystals as raw materials enables the production of target materials with higher purities than powder crystals. The film process can prevent the inclusion of impurities in the film crystals. This is likely to reduce the impurity level and improve the semiconducting properties of the CsPbBr_3_ films. In addition, the TRMC technique [27,34,36,40–48] was incorporated to investigate how the photoconductivity properties are modulated by the film crystallinity and evaluate the growth window. Herein, film materials can be deposited close to the thermodynamic equilibrium conditions at a certain temperature and pressure, thereby maximizing the functional properties [49–51]. The TRMC technique is a microwave pump-probe method, which is a non-contact evaluation method for quantifying the mobility and lifetime of carriers in semiconducting materials. In particular, these have been employed widely to evaluate the carrier transportation properties of organic semiconductors [47], solar cell materials [34,36,40–43,48], and photocatalysts [44–46]. TRMC measurements enable us to approximately evaluate the process dependence of the optical functionality obtained from the crystallinity without creating complex device structures composed of halide perovskite films. TRMC measurements have revealed that a CsPbBr_3_ film synthesized by PLD has a high effective mobility and long photocarrier lifetime (almost equivalent to that of a single crystal).

Another advantage of laser ablation is its applicability to device structures. To fabricate LED devices using halide perovskite films as light-emitting layers, the halide perovskite film is sandwiched between a hole transport layer (HTL) and an electron transport layer (ETL) [35,52,53]. Therefore, the characteristics of LED devices are affected significantly by the film crystallinity and mobility of both HTL and ETL films. When the film quality and mobility are inferior and low, respectively, the injection and transportation of holes or electrons are delayed. This, in turn, results in a decrease in the recombination efficiency with electrons or holes and, ultimately, a decrease in the light-emitting efficiency. In addition, charges would accumulate at the interface between the light-emitting layer and HTL or ETL films, thus deteriorating the interface structure [54–57]. However, if ETL and HTL films with high mobility are used, holes and electrons can be transported smoothly to the light-emitting layer. The resistance of the entire device is reduced, and the driving voltage is lowered. This improvement reduces the power consumption of LED devices and extends their life. Therefore, the film quality and mobility parameters of the ETL and HTL strongly affect the light-emitting efficiency, driving voltage, lifetime, and stability of LED devices. It is essential to improve the quality of the light-emitting layer of the halide perovskite film by PLD synthesis using a single-crystal target and to establish a vacuum process that can simultaneously synthesize ETL and HTL films with high crystallinity to improve device functions. In this study, we developed a system that can perform PLD and IR-MBE in a single vacuum chamber. We then fabricated a heterostructure of the light-emitting layer sandwiched between the ETL and HTL films by a laser ablation process combining a UV pulsed laser and an IR continuous-wave (CW) laser in dry vacuum to fabricate LED devices that emit green light.

## 2. Experimental section

CsPbBr_3_ thin films were deposited on an α-Al_2_O_3_(0001) substrate (Shinkosya, Japan) by a neodymium yttrium aluminum garnet (Nd:YAG) PLD (Vacuum Products: Double laser PVD, Japan). CsPbBr_3_ single crystals were used as the target material for PLD growth. These were synthesized using the ITC process [36–39]. The details are shown in Figure S1. A CsPbBr_3_ single-crystal target was ablated with the fourth harmonic (266 nm) pulses from a YAG laser (LOTIS LS-2145N/4, Belarus) at a pulse rate of 10 Hz and fluence of 0.9 J/cm^2^. The high repetition rate of 10 Hz was selected to suppress the evaporation of the volatile Cs element [58]. The sample holder was heated using a continuous-wave wave IR semiconductor laser with a wavelength of 808 nm [48,59]. The growth temperature was maintained at 150, 200, and 250°C. During the film growth, the film thickness and growth rate were monitored using a quartz crystal oscillator (QCM, INFICON: STM-2, Switzerland). The QCM was cooled down by the water flow during the film deposition. Moreover, the IR-laser heating method does not heat up the PLD chamber due to the high heat insulation, enabling us to keep the QCM temperature constant during the film deposition and precisely measure the film thickness.

The film orientation and lattice parameters were determined by Cu Kα X-ray diffraction (XRD, Rigaku Smartlab, Japan). The thicknesses of the films were measured using a stylus profiler (Kosaka Laboratory: ET200A, Japan). Scanning electron microscopy energy-dispersive X-ray spectroscopy (SEM-EDS, JEOL, JSM-IT200, Japan) was used to analyze the film stoichiometry. To mitigate the charging effect during EDS measurement, a perforated metal mask made of stainless steel was placed on the surface of the CsPbBr_3_ film [27,30]. The EDS spectra were measured from the area of 12.8 × 9.6 μm^2^. The accelerating voltage was 20 kV. The Cs/Pb ratio was calibrated by using commercial CsPbBr_3_ powder (Tokyo Chemical Industry Co., C3569, Japan).

TDS measurements (ESCO, TDS1200, Japan) were conducted on the CsPbBr_3_ powder to investigate the temperature-dependent thermal stability of the CsPbBr_3_ phase under vacuum. A quartz dish placed on a blackbody plate was used to hold 2 mg of the CsPbBr_3_ powder. It was then heated using an IR lamp. The sample temperature was increased from 50 to 320°C at a rate of 30 °C/min. Quadrupole mass spectrometry (QMS) was performed at each temperature during the heating process to identify the evaporated elements [27].

The optical transmittances of the CsPbBr_3_ films were analyzed using a UV-visible microspectrometer (JASCO, MSV-5700, Japan). PL measurements were performed using a custom-built fluorescence spectrometer. The film samples were excited at 410 nm using a continuous-wave semiconductor laser (Thorlabs LP405C1, Japan). The fluorescence spectra were collected via an optical fiber using a charge-coupled device (CCD) linear image sensor (Hamamatsu: PMA-12, Japan). The luminescence was detected from the irradiated spot with a diameter of 280 μm. The pulse intensity was tuned using neutral density (ND) filters ranging from 0.0038 to 3.6 W/cm^2^. The time-resolved PL (TRPL) was measured using time-correlated single-photon counting (TCSPC). A pulsed 375 nm laser diode (Edinburgh Instruments, EPL-375, UK) was used as the excitation source. The emissions were collected using a cooled high-speed photomultiplier tube detector head for photon counting (Becker & Hickl GmbH: PMC-150–04, Germany).

For the TRMC measurements, microwaves generated using an oscillator based on a 200 mW Gunn diode (Nakadai MGS-15B, Japan) were used as the probe [27,44,45]. The frequency was tuned from 8 to 9 GHz using a varactor diode. The microwaves were directed toward the sample cavity through a waveguide. The third harmonic (355 nm) of an Nd:YAG laser (VM-TIM, VM-200–10-ST, Germany) was used for the UV excitation measurements. The laser pulse duration was 10 ns. The pulse intensity was tuned using ND filters ranging from 7.3 × 10^−4^ to 1.8 *μ*J/cm^2^. The reflected microwaves were detected using a diode (NEC, IN23WE, Japan). The measured signals were processed using a digital oscilloscope (TDS680C, Tektronix, USA) and analyzed on a computer. The TRMC measurements were performed under identical excitation conditions. Therefore, the magnitude of the TRMC signal could be compared with that of the reference anatase TiO_2_ nanocrystalline film sample [44,45]. Thus, the effective mobility was evaluated. For the TRMC measurements of CsPbBr_3_ powder (Tokyo Chemical Industry Co., C3569, Japan) and single crystals, the measurement samples were inserted into a quartz tube and fixed in the cavity. For insertion, a 1 cm-square CsPbBr_3_ single crystal was cut into millimeter-sized pieces.

To fabricate LED devices, a light-emitting CsPbBr_3_ layer was sandwiched between an Mg_0.3_Zn_0.7_O film as an ETL and an *N,N*’-Di-1-naphthyl-*N,N*’-diphenylbenzidine (α-NPD, Tokyo Chemical Industry Co., D5126, Japan) film as an HTL. The heterostructures were fabricated on ITO/glass substrates (GEOMATEC Co., Ltd. No. 1005, Japan). Through a lithography process, 300 nm-thick ITO films were arranged on a 1 cm-square glass substrate with a line pattern of 0.1 ×1 cm^2^ and spaced 0.5 mm apart. A 50 nm-thick Mg_0.3_Zn_0.7_O film was deposited as an ETL on the entire surface of a specially processed indium tin oxide (ITO)/glass substrate via PLD [60–62]. During deposition, the oxygen pressure was maintained at 1 ×10^−5^ Torr, and the substrate temperature was maintained at 200°C. As the second layer, a 240 nm-thick CsPbBr_3_ film was deposited by PLD at 200°C. As the third layer, a 50 nm-thick α-NPD film was deposited as a HTL by IR-MBE at room temperature. The α-NPD powder raw material (Tokyo Chemical Industry Co.: D5126, Japan) was mixed with Si powder and ablated with quasi-pulse light of 808 nm IR laser to deposit the α-NPD film. The mixture ratio of the silicon powder was 50 wt %. This was optimized based on previous studies [27]. As the fourth layer, a 0.75 nm-thick MoO_x_ film was deposited under vacuum by PLD. Finally, a 100 nm-thick Au film was deposited as the top electrode in a line shape of 0.1 ×1 cm^2^ using an electron-beam evaporation process through a contact stencil mask. Aluminum wire bonding was used to contact the top and bottom electrodes. Current – voltage (I-V) measurements were performed using a Keithley 2450 SMU source meter (USA). The junction polarity is defined as positive when a positive voltage is applied to the top Au electrode. Electroluminescence (EL) spectra were recorded in an electric field using a CCD linear image sensor (Hamamatsu: PMA-12, Japan).

## 3. Results and discussion

### 3.1. Structural analysis of CsPbBr_3_ films

**Figure 1.**
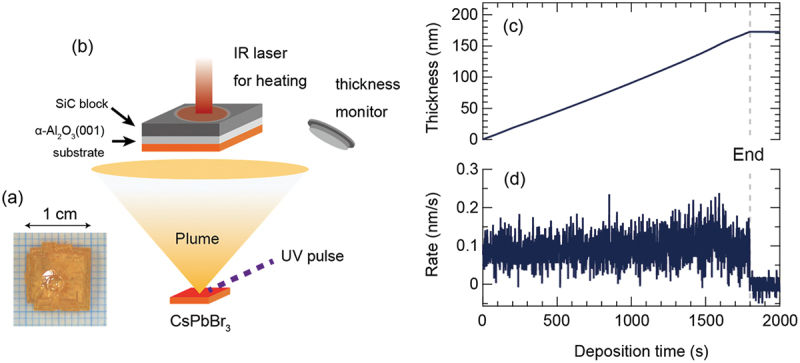
(a) The photograph of a CsPbBr_3_ single crystal target used for the PLD process. The circle on the crystal surface denotes the area ablated by UV laser pulses. (b) Schematic illustration of a PLD system for the growth of CsPbBr_3_ thin films deposited on α-Al_2_O_3_ (0001) substrate. (c) and (d) show the CsPbBr_3_ film thickness and growth rate, which were monitored during the CsPbBr_3_ film growth. The film thickness shown in (b) increased linearly as a function of the deposition time. The deposition rate was maintained at 0.1 nm/s during the entire deposition.

A 1 ×1 cm^2^ CsPbBr_3_ single crystal was grown by repeating the ITC process three times. Figure 1(a) shows a photograph of the CsPbBr_3_ single crystal. The crystal was placed on a target holder and fixed inside the PLD chamber, as illustrated in Figure 1(b). The single-crystal target was rotated and ablated with a 266 nm Nd:YAG pulsed laser at a frequency of 10 Hz to evaporate the CsPbBr_3_ raw material. A CsPbBr_3_ film was deposited on the α-Al_2_O_3_(0001) substrate heated by an IR semiconductor laser [49,59]. During the film deposition, the substrate temperature was maintained at 150°C, 200°C, 250°C, and 300°C. At 300°C, no CsPbBr_3_ film was deposited on the α-Al_2_O_3_(0001) substrate.

Figure 1(c) and (d) shows the thickness (c) and growth rate (d) of the CsPbBr_3_ film monitored during deposition. By irradiating the CsPbBr_3_ single crystal with a pulsed laser operating at 10 Hz, the CsPbBr_3_ film was deposited at a deposition rate of approximately 0.1 nm/s. The result was the deposition of a 170 nm-thick CsPbBr_3_ film. This implies that the thickness of the CsPbBr_3_ film can be controlled within 0.01 nm per laser pulse. Precise thickness control is one of the advantages of the vacuum film process over the solution-coating method, which is commonly used for synthesizing halide perovskites. It enables the tuning of the thickness of heterostructures (such as solar cells and LED devices) to any desired value.

**Figure 2.**
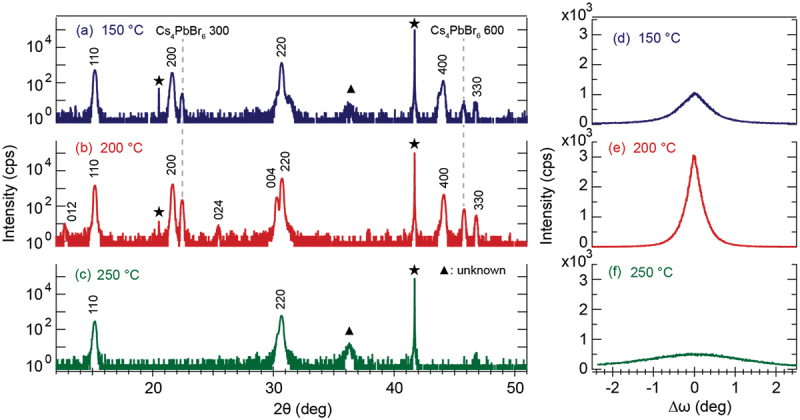
XRD patterns of CsPbBr_3_ films grown at 150°C (a, d), 200°C (b, e), and 250°C (c, f). The symbols ‘★’ and ‘▲’ in (a-c) denote the substrate and unknown phase. The rocking curves in (d-f) were measured for the CsPbBr_3_ 220 reflection. The FWHM values were calculated at 0.84° (d), 0.42° (e), and 3.28° (f).

Figure 2(a–c) shows the XRD patterns of the CsPbBr_3_ films deposited at 150°C, 200°C, and 250°C. Polycrystalline CsPbBr_3_ thin films with orthorhombic phase structures were grown on α-Al_2_O_3_(0001) substrates. The XRD patterns of CsPbBr_3_ films deposited at 150°C and 200°C show the precipitation of an impurity Cs_4_PbBr_6_ layer in addition to the precipitation of the CsPbBr_3_ phase. During IR-MBE growth, the optical properties deteriorated because of the precipitation of the impurity CsPb_2_Br_5_ phase with a Cs deficiency [27]. In contrast, PLD using a pulsed UV laser suppressed the formation of Cs deficiencies by instantaneously evaporating the raw material with a nanosecond UV laser. This yielded an improved CsPbBr_3_ film crystallinity.

To compare the crystallinity of the CsPbBr_3_ films prepared at three substrate temperatures, Figure 2(d–f) shows the rocking curves of the CsPbBr_3_ 220 reflection. The full width at half maximum (FWHM) values of the three films were estimated at 0.84°, 0.42°, and 3.28°, respectively. The CsPbBr_3_ film deposited at 200°C had the best crystallinity. This result is strongly associated with the peak intensity of the CsPbBr_3_ phase in the XRD patterns shown in Figure 2(a–c).

**Figure 3.**
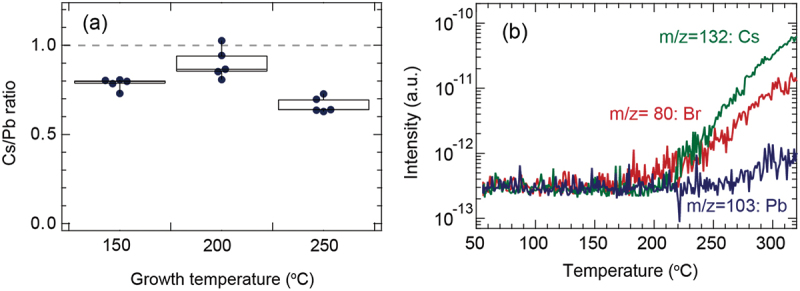
(a) Box plots of the growth temperature dependent Cs/Pb ratio in CsPbBr_3_ films. The grey dotted line denotes the chemical composition of the commercial CsPbBr_3_ powder. (b) TDS spectra of m/z = 132 (Cs), 80 (br), and 103 (Pb) during heating from 50 to 320°C at a rate of 30 °C/min.

To investigate why the CsPbBr_3_ film deposited at 200°C has the highest crystallinity, the chemical composition of the CsPbBr_3_ film was analyzed using SEM-EDS. Figure 3(a) shows the box plot of the growth temperature dependent Cs/Pb ratio of the CsPbBr_3_ films. The average Cs/Pb ratio of the CsPbBr_3_ film deposited at 200°C (which had the highest crystallinity) was 0.90. This was closest to that of the bulk CsPbBr_3_ powder. The deposition temperature was close to the growth window, where the chemical composition of the film was tuned spontaneously to the optimal composition of the bulk phase [49–51]. Meanwhile, the Cs/Pb values of the CsPbBr_3_ films deposited at 150°C and 250°C were estimated to be 0.78 and 0.67, respectively. This indicated the formation of Cs deficiencies. XRD measurements of the CsPbBr_3_ films deposited at 150°C and 250°C showed that the FWHM of the rocking curves broadened and that the XRD intensity was small (Figure 2). In particular, when a CsPbBr_3_ film was deposited at the temperature range above 200°C, the Cs deficiency was observable.

To investigate the origin, Figure 3(b) shows the TDS spectrum when the temperature was increased from 50 to 320°C at a heating rate of 30 °C/min. A TDS analysis was used to clarify which elements of CsPbBr_3_ evaporate within a temperature range in vacuum. A tendency for Cs and Br to evaporate was observed from approximately 200°C. However, Pb (which has a high vapor pressure) is less likely to be depleted. Although the surface diffusion during growth is likely to be promoted at higher temperatures, Cs evaporates during the deposition of CsPbBr_3_ films. This causes Cs deficiency when the film is deposited above 200°C, resulting in the deterioration of the CsPbBr_3_ film crystallinity.

### 3.2. Optical absorption and luminescence analysis of CsPbBr_3_ films

**Figure 4.**
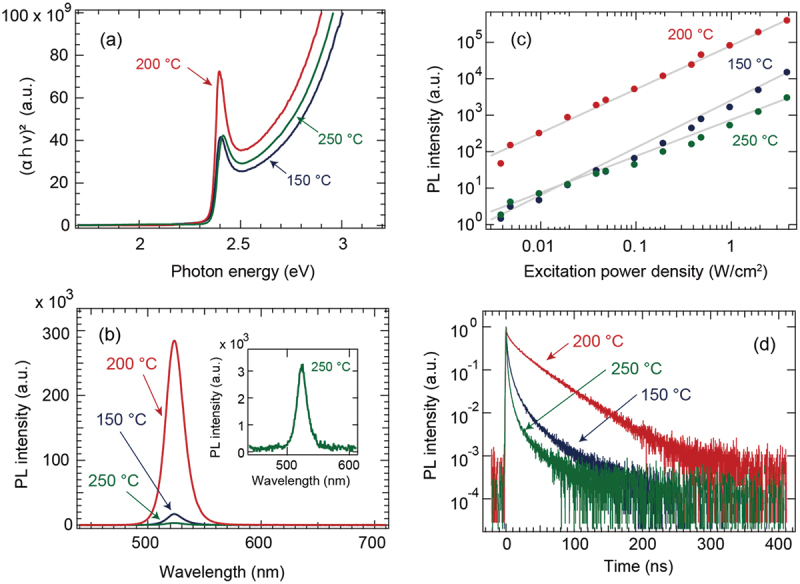
(a) Tauc plots, (b) PL spectra, (c) excitation laser power density dependence on PL emission intensity at 524 nm, and (d) transient PL profiles of the CsPbBr_3_ thin films grown at 150°C (blue), 200°C (red), and 250°C (green). The inset in (b) magnifies the PL spectrum of the CsPbBr_3_ film grown at 250°C. The gray line in (c) denotes the fitting line by using the power law.

Figure S2 and Figure 4(a) show the optical absorption spectra and the Tauc plot, respectively, estimated from the optical transmittance of CsPbBr_3_ films deposited at 150°C, 200°C, and 250°C. The band gap value of all the films was calculated at 2.36 eV. This corresponds to the value reported in the literature [23,24]. A strong exciton absorption peak was observed 2.4 eV above the bandgap. This indicated that the exciton is stable at room temperature. The stability of the exciton is also confirmed by the relatively large exciton binding energy of 33 meV in CsPbBr_3_ films [63]. In addition, the CsPbBr_3_ film deposited at 200°C (which displayed the best crystallinity according to the XRD analysis) had the highest exciton optical absorption. This indicates that the density of impurities and defects that scatter excitons reduces with an improvement in crystallinity [64]. Therefore, the exciton lifetime and PL emission are likely to be longer and higher, respectively, with improved crystallinity.

Figure 4(b) shows the PL spectra measured for the CsPbBr_3_ films deposited at 150°C, 200°C, and 250°C when the excitation wavelength was 410 nm. A strong emission peak was observed at 524 nm for all the CsPbBr_3_ films. The emission wavelength is consistent with the optical bandgap of each CsPbBr_3_ film, as shown in Figure 4(a). Comparing the PL intensity, the CsPbBr_3_ film deposited at 200°C had the strongest PL intensity of 2.8 × 10^5^ counts, whereas the CsPbBr_3_ films deposited at 150°C and 250°C showed 1.7 × 10^4^ and 3.3 × 10^3^ counts, respectively. When the relative emission quantum yield of the CsPbBr_3_ film deposited at 200°C was estimated at 100, those of the other samples deposited at 150°C and 250°C were 6.1 and 1.2, respectively. The PL emission intensity decreased dramatically as the film crystallinity was worse.

Figure 4(c) shows the excitation intensity dependence of the PL emission intensity at 524 nm for CsPbBr_3_ films deposited at 150°C, 200°C, and 250°C when the excitation wavelength was 410 nm. The dependence on excitation light intensity is effective for clarifying the relevant recombination pathways of the photogenerated carriers in the material [65]. Because the PL emission intensity experimentally increases proportionally to a power law (I_PL_ ∝ I_exc_^α^), the power factor α is a parameter that reflects the carrier recombination process present. The PL intensity of all the CsPbBr_3_ films deposited at 150°C, 200°C, and 250°C responded linearly to the excitation light intensity, and no shift of the emission peak was observed. The α values of the CsPbBr_3_ films deposited at 150°C, 200°C, and 250°C were estimated to be 1.3, 1.2, and 1.0, respectively (approaching α = 1). A significant nonlinear trend owing to recombination influenced by free carriers in the defect states is not observed in Figure 4(c).

Figure 4(d) shows the TRPL profiles measured by TCSPC for CsPbBr_3_ films deposited at 150°C, 200°C, and 250°C. The TRPL profile of the CsPbBr_3_ film deposited at 200°C shows a linear variation in PL intensity on a logarithmic graph, whereas the other two profiles do not show a linear profile. In general, the PL transient profile can be fitted using the following exponential function with a time constant:

1 $$I_{\mathrm{PL}}=A\exp\left\{-{\left(\frac{t}{\tau }\right)}^{\alpha }\right\},$$

where t is the time, τ is the emission lifetime, A is the emission intensity, and α is the distribution parameter [66,67]. If α = 1, Eq. 1 degenerates to a single exponential, and the heterogeneity is negligible. If α deviates from one, it implies that heterogeneity exists. The α values for the CsPbBr_3_ films deposited at 150°C, 200°C, and 250°C are estimated at 0.35, 0.6, and 0.3, respectively. The profile of the CsPbBr_3_ film deposited at 200°C is closest to a single exponential profile. This indicates that the multiple recombination was suppressed.

The half-lives of the PL transient profiles were calculated at 1.0 ns, 7.2 ns, and 0.51 ns. These correlate with the PL emission intensity results in Figure 4 (b) and (c). The CsPbBr_3_ film deposited at 200°C showed the longest emission lifetime. Meanwhile, those deposited at the other two temperatures had more crystal defects, as indicated by the structural evaluation by XRD. These trapped the generated photocarriers at the defect levels and shortened the emission lifetime by nonradiative recombination.

The TRPL profile in Figure 4(d) clearly reveals that the shape and lifetime of the TRPL profiles are strongly dependent on the CsPbBr_3_ film crystallinity, indicating that the PL of the CsPbBr_3_ film is strongly dominated by the exciton emission. The CsPbBr_3_ films deposited at 150°C and 250°C were so defective that the exciton emission efficiency was significantly decreased. In contrast, the CsPbBr_3_ film deposited at 200°C, which had the best crystallinity, showed a high exciton optical absorption and high luminescence efficiency.

### 3.3. TRMC analysis of CsPbBr_3_ films

We investigated the deposition temperature dependence of the photoconductivity of CsPbBr_3_ films by TRMC measurement. This is a transient absorption spectroscopy technique that measures the electrical conductivity (σ) of charge carriers generated in a thin film sample by UV laser irradiation from the amount of microwave absorption [27,34,36–38,40–46]. TRMC was used as an effective technique to characterize the dynamics of charge carriers in various halide perovskite films and single crystals [27,34,36–38,40–43].

The absorption of microwave power (-∆P/P) can be expressed as

2 $$-\left(\frac{\Delta P}{P}\right)=-K\sigma =K^{\prime }N_{e}\sum \mu$$

where K and $K^{\prime }$ are constants, $N_{e}$ is the number of charges present, and ∑μ is the sum of the mobilities of the positive and negative charge carriers. The TRMC transient signal contains information regarding the amount and mobility of charge carriers in the film sample, as well as the kinetics of electron and hole recombination and charge carrier trapping. Immediately after photoexcitation with a pump UV laser, $N_{e}$ attains a maximum value and represents the mobile charge generation efficiency ($\Phi_{\mathrm{CG}}$). If the wavelength of the excitation light is absorbed completely by the thin-film crystal, Eq. 2 can be expressed as follows:

3 $$-\frac{{\left(\frac{\Delta P}{P}\right)}_{\max}}{I_{\mathrm{ex}}}=K^{\prime \prime }\phi_{\mathrm{CG}}\sum \mu ,$$

where $K^{\prime \prime }$ is a constant and $I_{\mathrm{ex}}$ is the intensity of the UV laser. Therefore, the TRMC technique allows for the contactless estimation of the effective charge mobility, which varies significantly with the crystallinity of the halide perovskite thin films. Another significant advantage of TRMC is that it provides information on the dynamics associated with the trapping and recombination of photoinduced charge carriers. The decay curve of the TRMC signal indicates the photocarrier lifetime. For example, a TRMC analysis of CsPbI_3_ films has shown that thin films with fewer crystal defects exhibit longer photocarrier lifetimes, whereas those with higher trap densities exhibit shorter photocarrier lifetimes [34]. Therefore, TRMC evaluation enables a comparison of the electrical properties of halide perovskite films synthesized by different film processes without the deposition of metal electrodes.

**Figure 5.**
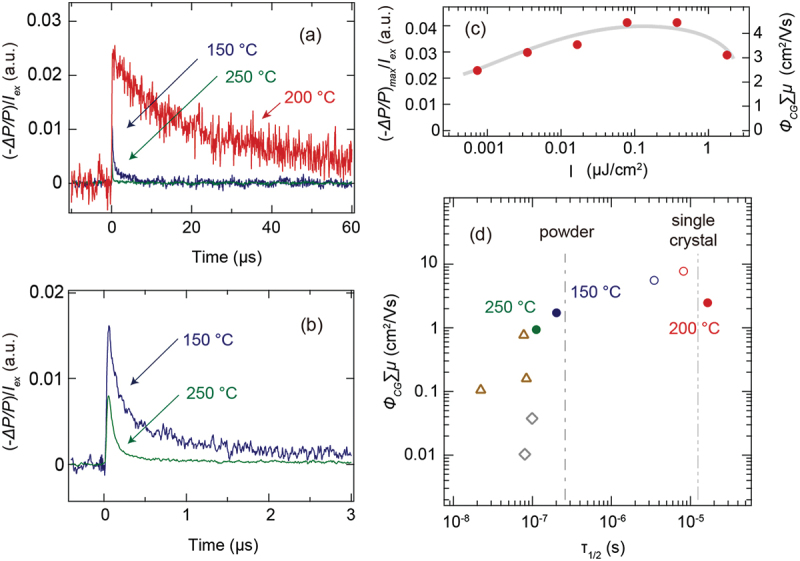
(a) and (b) transient profiles of the $\left(\left(-\mathrm{\Delta P}/P\right)/I_{\mathrm{ex}}\right)$ signals for CsPbBr_3_ films grown at 150°C (blue), 200°C (red), and 250°C (green). (c) The $\left({\left(-\mathrm{\Delta P}/P\right)}_{\max}/I_{\mathrm{ex}}\right)$ (left axis) and $\Phi_{\mathrm{CG}}\sum \mu$ (right axis) as functions of the excitation laser intensity of the CsPbBr_3_ film grown at 200 ℃. (d) The $\Phi_{\mathrm{CG}}\sum \mu$ (closed circle) of the CsPbBr_3_ films as a function of τ_1/2_. For comparison, the brown triangles and grey diamonds plot the TRMC results for the CsPbBr_3_ thin films in references [40,41]. The grey dotted and solid lines denote the measurement data of the CsPbBr_3_ powder and single-crystal samples, respectively. The open circles denote the measurement data to check the reproducibility of the TRMC properties for CsPbBr_3_ films grown at 150°C (blue) and 200°C (red).

The TRMC technique was applied to CsPbBr_3_ films deposited by PLD to measure their effective mobility and photocarrier lifetime. Figure 5(a) shows the transient $\left(\left(-\mathrm{\Delta P}/P\right)/I_{\mathrm{ex}}\right)$ profiles of CsPbBr_3_ films deposited at 150°C (blue), 200°C (red), and 250°C (green). The TRMC profiles were measured at laser energies of 3.48, 0.73, and 16.57 nJ/cm^2^. Each energy corresponds to the smallest excitation laser energy density to minimize the secondary hole-electron recombination. The magnitude of the TRMC signal is strongly affected by the film crystallinity. Particularly for the CsPbBr_3_ film with poor crystallinity, the TRMC signal is so weak that the excitation laser energy density had to be increased to detect the TRMC signal. Consequently, the excitation laser energy was varied for each sample, affecting the noise level of the TRMC signals for each profiles in Figure 5(a).

Mobile carriers were generated in the sample by irradiation with a 355 nm pulsed laser pump light. These absorbed the microwave irradiated as a probe light. As a result, $\left(\left(-\mathrm{\Delta P}/P\right)/I_{\mathrm{ex}}\right)$ increased abruptly at 0 μs when the pulsed laser was irradiated on the film sample. The $\left(\left(-\mathrm{\Delta P}/P\right)/I_{\mathrm{ex}}\right)$ in all the samples decreased gradually owing to the trapping and recombination processes. Here, the half-life (*τ*_*1/2*_) of $\left(\left(-\mathrm{\Delta P}/P\right)/I_{\mathrm{ex}}\right)$ is defined as the lifetime of the photocarriers. The *τ*_*1/2*_ of the CsPbBr_3_ sample deposited at 200°C (which had the best crystallinity in the XRD measurements) was 16.5 μs. Meanwhile, the other two films with low crystallinity were observed to have significantly shorter *τ*_*1/2*_. Figure 5(b) shows the transient $\left(\left(-\mathrm{\Delta P}/P\right)/I_{\mathrm{ex}}\right)$ profiles of the two samples measured precisely on a short time scale. The *τ*_*1/2*_ of the CsPbBr_3_ thin films deposited at 150°C and 250°C were estimated to be 0.20 μs and 0.11 μs, respectively. These are approximately two orders of magnitude shorter than that of the CsPbBr_3_ film deposited at 200°C.

Furthermore, the $\left({\left(-\mathrm{\Delta P}/P\right)}_{\max}/I_{\mathrm{ex}}\right)$ of the CsPbBr_3_ film deposited at 200°C was 0.023, whereas those of the CsPbBr_3_ thin films deposited at 150°C and 250°C were 0.016 and 0.009, respectively. The main difference between the process conditions of the three samples was in the deposition temperature. It resulted in a significant difference in the crystallinity of the CsPbBr_3_ films (Figure 2). As the crystallinity deteriorated, the defect concentration increased. As a result, the generation efficiency or the mobility of the mobile charges decreased, thereby reducing the intensity of $\left({\left(-\mathrm{\Delta P}/P\right)}_{\max}/I_{\mathrm{ex}}\right)$.

Figure 5(c) shows the intensity of $\left({\left(-\mathrm{\Delta P}/P\right)}_{\max}/I_{\mathrm{ex}}\right)$as a function of $I_{\mathrm{ex}}$ for the CsPbBr_3_ film deposited at 200°C. When the excitation light intensity is high, $\left({\left(-\mathrm{\Delta P}/P\right)}_{\max}/I_{\mathrm{ex}}\right)$ generally becomes small owing to the secondary effect of hole – electron recombination. As the excitation light intensity decreases, the influence of the secondary effect weakens and $\left({\left(-\mathrm{\Delta P}/P\right)}_{\max}/I_{\mathrm{ex}}\right)$ increases. Finally, at the minimum excitation light intensity, $\left({\left(-\mathrm{\Delta P}/P\right)}_{\max}/I_{\mathrm{ex}}\right)$ saturates at a certain value and attains a maximum value. However, in Figure 5(c), $\left({\left(-\mathrm{\Delta P}/P\right)}_{\max}/I_{\mathrm{ex}}\right)$ increases as the excitation light intensity decreases from 1.78 *μ*J/cm^2^ to 0.079 *μ*J/cm^2^, and decreases as it decreases from 0.079 *μ*J/cm^2^ to 0.00073 *μ*J/cm^2^. This reveals that the $I_{\mathrm{ex}}$ dependence is bell-shaped. A similar bell-shaped excitation light intensity trend has been reported for anatase TiO_2_ films [44,45]. It is attributed to the rapid immobilization of free carriers at the defect levels, which reduces the yield of free charges at low excitation densities. The bell-shape was not seen for the CsPbBr_3_ film deposited on the same α-Al_2_O_3_(0001) substrate by IR-laser MBE [27], which can gently deposit the films by the thermal evaporation due to the heating by IR-laser irradiation. On the other hand, in the PLD process, the raw material is converted into plasma by UV laser ablation. The plasma energy varies depending on the magnitude of the UV laser energy density used for ablation [60,68,69]. In the case of the oxide film deposition using Nd:YAG laser, the plasma energy can reach 100 eV. As a result, the high-energy plasma ablates the already deposited film surface, generation point defects in the film and causing deterioration of the film properties. Therefore, the bell shape in Figure 5(c) is attributed to the PLD process using the Nd:YAG laser.

The TRMC measurement has certain advantages that enable us to quantitatively compare the effective mobility and *τ*_*1/2*_ of the films with those in the literature. The right axis of Figure 5(c) shows the normalized effective mobility of $\Phi_{\mathrm{CG}}\sum \mu$ by using the reference sample of anatase TiO_2_ nanocrystalline film [44,45]. When excited at a low energy intensity that suppresses the recombination of holes and electrons, the $\Phi_{\mathrm{CG}}\sum \mu$ of the CsPbBr_3_ film deposited at 200°C was calculated at 2.47 cm^2^/Vs, whereas those for 150°C and 250°C were 1.18 and 0.93 cm^2^/Vs, respectively. Figure 5(d) maps out $\Phi_{\mathrm{CG}}\sum \mu$ as a function of the *τ*_*1/2*_ of the TRMC signal to compare the TRMC characteristics that depend on the film synthesis process. In addition to the experimental results for the three film samples prepared by PLD, the reported values [40,41] of the CsPbBr_3_ films prepared by the solution-coating process are mapped in Figure 5(d). In general, a film sample with good crystallinity should exhibit the higher $\Phi_{\mathrm{CG}}\sum \mu$ and longer τ_1/2_ of the TRMC signal, which is extended by suppressing the non-radiative recombination transitions. The properties of highly crystalline CsPbBr_3_ films are plotted on the upper right. The CsPbBr_3_ film synthesized by PLD obtained an improved crystallinity through a vacuum process using a single crystal target. This yielded the higher $\Phi_{\mathrm{CG}}\sum \mu$ and longer τ_1/2_ values. This was also reflected by the fact that the impurity concentration of the CsPbBr_3_ film was minimized. In particular, the sample observed at 200°C had the best crystallinity, which suppressed non-radiative recombination at defect levels. Thus, it displayed the highest $\Phi_{\mathrm{CG}}\sum \mu$ and longest τ_1/2_.

To further investigate whether the τ_1/2_ in the TRMC signals was owing to the film crystallinity, we performed TRMC measurements on commercial CsPbBr_3_ powder and a CsPbBr_3_ single crystal. The transient profiles of the TRMC signals are shown in Figure S3 (a) and (b). Both the decay profiles are similar to those of the CsPbBr_3_ film samples, as shown in Figures 5(a) and (b). The τ_1/2_ values of the CsPbBr_3_ powder and single crystal samples were calculated to be 0.258 μs and 13.4 μs, respectively. As previous film data indicate, the $\Phi_{\mathrm{CG}}\sum \mu$ and τ_1/2_ values increased with an increase in the crystallinity of the sample. For the powder and single crystal samples, the $\Phi_{\mathrm{CG}}\sum \mu$ values cannot be compared quantitatively because the sample shape was different from those of the film samples. Therefore, only the τ_1/2_ values obtained from the TRMC profiles are plotted in Figure 5(d) and compared with the data of the other film samples. The τ_1/2_ of the CsPbBr_3_ powder was almost consistent with those of the CsPbBr_3_ films prepared by the solution-coating process and those prepared by PLD at 150°C and 250°C. Meanwhile, the single crystal sample showed the longer τ_1/2_. It was almost equivalent to that of the CsPbBr_3_ film prepared at 200°C by PLD. A longer lifetime of 100 μs at room temperature has been reported for CsPbBr_3_ single crystals [36]. A longer τ_1/2_ of the TRMC signal is likely by improving the quality of CsPbBr_3_ single crystals. This result, in which the photocarrier lifetime varies significantly in CsPbBr_3_ films with different crystallinities, indicates that photoexcited hot carriers are trapped at deep defect levels and lost by non-radiative recombination. This is consistent with a report on CsPbBr_3_ nanocrystals [1,4]. This indicates that CsPbBr_3_ films, which have a large band gap compared with iodine-based halide perovskites, are more susceptible to the generation of defects.

In order to investigate the reproducibility of TRMC properties, the CsPbBr_3_ films were prepared by the same procedure, again. Figure S4 (a-c) show the XRD patterns of CsPbBr_3_ films grown at 150°C, 200°C and 250°C. The best crystallinity was seen for the CsPbBr_3_ film grown at 200°C. At 250°C, CsPbBr_3_ phase was not crystallized. Figure S4 (d) shows the transient TRMC profiles of the CsPbBr_3_ films grown at 150°C, 200°C and 250°C. The largest TRMC signal and longest photocarrier lifetime was observed for the CsPbBr_3_ film grown at 200°C. For CsPbBr_3_ film grown at 250°C, the TRMC signal was not seen due to no crystallization of CsPbBr_3_ phase. In Figure 4(d), the open circles denote the plots to check the reproducibility of CsPbBr_3_ films grown at 150°C and 200°C. The best growth temperature of 200°C is reproducible to improve the CsPbBr_3_ film crystallinity and maximize the TRMC properties of CsPbBr_3_ films grown via PLD.

The photocarrier lifetimes observed in the TRMC measurements were compared to those observed in the TRPL measurements, as shown in Figure 4(d). The lifetime of the TRMC profile in Figure 5(a) is three orders of magnitude longer than that of the TRPL signal. A large difference between the TRPL and TRMC measurements was observed for the CH_3_NH_3_PbI_3_ single crystals [43] and CsPbI_3_ films [34]. TRPL measures the lifetime of the radiative recombination of electrons and holes. Especially for CsPbBr_3_ films, the PL characteristics are strongly dominated by excitons, as can be clearly seen in Figure 4(a-d) . That is, the PL lifetime depends on how long the excitons survive to emit light. Meanwhile, TRMC measurements detect the product of the mobility and generation of free carriers (consisting of holes and electrons) in a time-resolved manner (Eq. 3). TRMC measurement can directly detect no signal originated from excitons, which are electrically neutral. Therefore, TRMC measurements can monitor the presence and mobility of the free carriers. That is, it indicates the length of the carriers in the mobile state. Therefore, TRMC and TRPL measurement techniques examine different carrier dynamics, thus resulting in significantly different lifetimes. The advantage of TRMC over TRPL is that it is sensitive to nonradiative recombination because the observed signal is attenuated if mobile carriers are lost. This is regardless of whether the recombination is radiative or nonradiative. In addition, the behaviour of mobile carriers was observed to be contact-free throughout the sample, which made these vulnerable to the bulk and defects. Therefore, mapping (as shown in Figure 5(d)) is effective for comparing the optical functions of thin films, which are highly process-dependent. This indicates that there is no defect tolerance in the CsPbBr_3_ films.

### 3.4. LED devices using CsPbBr_3_ films

**Figure 6.**
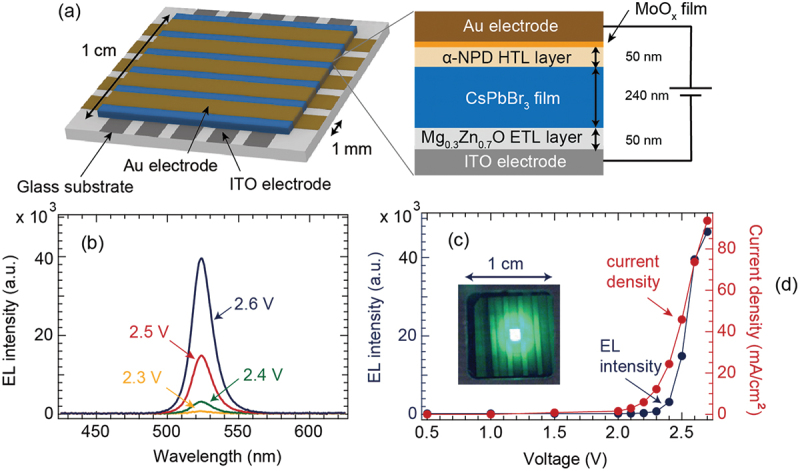
(a) Schematic illustration, (b) EL spectra and (c) EL intensity at 524 nm and the current density as a function of the voltage of the EL device composed of CsPbBr_3_ film. The inset shows the photograph of the EL device operated at 2.5 V. it presents a uniform green emission.

As shown in Figure 6(a), a heterostructure consisting of five layers of Au/MoO_x_/α-NPD/CsPbBr_3_/Mg_0.3_Zn_0.7_O was fabricated on an ITO/glass substrate by a vacuum process of PLD and IR-MBE. A striped ITO electrode was fabricated on a glass substrate using lithography. A CsPbBr_3_ film sandwiched between the MoO_x_/α-NPD HTL and Mg_0.3_Zn_0.7_O ETL was deposited on top of the ITO electrode. The electron affinity and ionization energy of the CsPbBr_3_ film have been reported to be 3.65 eV and 5.97 eV, respectively [9]. A 50 nm-thick Mg_0.3_Zn_0.7_O film was used as the ETL layer to inject electrons into the CsPbBr_3_ film [60–62]. The electron affinity of ZnO is 4.1 eV. However, it can be reduced to 3.5 eV by doping with 30% Mg [61,70]. Mg doping facilitates the injection of electrons into the light-emitting layers of LED devices. Heterostructures with Mg_0.3_Zn_0.7_O films having low electron affinity as the ETL are also used in organic solar cells [71] and perovskite LEDs [53] to reduce the interfacial barrier and improve the properties of photovoltaic devices.

As the second layer, a 240 nm-thick CsPbBr_3_ film was deposited at 200°C by PLD on a Mg_0.3_Zn_0.7_O ETL to serve as a light-emitting layer. As the third layer, a 50 nm-thick α-NPD film HTL was deposited by IR-MBE. The ionization energy of α-NPD film has been reported to be 5.35 eV [72]. This is lower than that of CsPbBr_3_ (5.97 eV), which facilitates hole injection. In addition, a 0.75 nm-thick MoO_x_ layer was deposited by PLD to improve the hole injection efficiency [35]. Finally, a 100 nm-thick Au film was deposited using electron beam deposition to function as the top electrode. A positive bias was applied at the top of the Au electrode.

Figure S5 (a) presents the optical transmittance of the LED device. For the ITO/glass substrate, the optical absorption increased at approximately 300 nm, and the transmittance reduced significantly. Furthermore, a fringe pattern owing to an ITO film thickness of 300 nm was observed in the transmission spectrum of the ITO film. Meanwhile, in the LED device sample, a four-layer heterostructure of MoO_x_/α-NPD/CsPbBr_3_/Mg_0.3_Zn_0.7_O was deposited on the ITO bottom electrode. The optical absorption originating from the CsPbBr_3_ light-emitting layer occurred at approximately 524 nm. Figure S5 (b) presents the PL spectrum of the heterostructure measured at an excitation wavelength of 410 nm. A strong PL emission was observed at 524 nm. It was verified that the light absorption edge and PL emission wavelength of the heterostructure of the CsPbBr_3_ film deposited on the glass substrate were almost consistent with the results for the CsPbBr_3_ film on the α-Al_2_O_3_(0001) substrate shown in Figure 4.

Figure 6(b) shows the EL spectrum measured when a voltage was applied. Around 2.3 V (which corresponds to the bandgap of the CsPbBr_3_ film), the current flowing through the device increased, and an EL emission was observed. As the voltage increased from 2.3 V to 2.6 V, the EL emission intensity increased. The half width of the EL spectrum was significantly narrow (16.5 nm). This indicated a high color purity. The inset of Figure 6(c) shows a photograph of the EL sample when a voltage of 2.5 V was applied. The entire device (with a size of 1 ×1 mm^2^) emitted a uniform green light. Figure 6(c) shows the EL emission intensity and current as functions of the applied voltage. The current exponentially increased from approximately 2 V, which is near the bandgap. As the current increased, the emission intensity increased in proportion to the magnitude of the current. The 2.3 V threshold and 524 nm EL emission wavelengths are consistent with the band gap of the CsPbBr_3_ film. This demonstrates that light is emitted by the recombination of electrons and holes in the CsPbBr_3_ film.

As is shown in Figure 2, we note that the CsPbBr_3_ films are contaminated with the Cs_4_PbBr_6_ phase, which is known to have higher luminescence properties than the CsPbBr_3_ phase [73,74]. XRD and EDS analyses have revealed that the volume of the CsPbBr_3_ phase is much smaller than that of the CsPbBr_3_ phase, implying that measurement results from Figures 4 to 6 are strongly dependent on the CsPbBr_3_ phase. However, the presence of the Cs_4_PbBr_6_ phase may have some effects on the PL, TRMC and EL properties.

The vacuum process using laser ablation creates heterostructures between halide perovskite materials and various compounds. In this study, we demonstrated the fabrication of LED devices by forming heterointerfaces between halide perovskite materials and the oxide Mg_0.3_Zn_0.7_O or organic material α-NPD. The formation of heterostructures is not limited to LED devices. It is also an effective means of energy production devices such as solar cell devices and photocatalysts. By improving the film quality of all structures through laser ablation, the mobility of each layer can be improved. This would yield enhanced device characteristics. Furthermore, the electronic structure at the heterostructure interface governs the characteristics of such devices. Novel functions that can be realized by forming artificial interfaces are well known in the material systems of graphene [75] and oxide [76] materials. Even in halide perovskite materials, the realization of heterointerfaces of different materials can yield many novel functions that cannot be achieved using the solution-coating process. Laser ablation is likely to realize a new heterointerface controlled at the atomic level. It can potentially become a process technology that would play a key role in the development of new devices composed of halide-based materials.

## 4. Conclusion

We fabricated heterostructures of halide perovskite CsPbBr_3_ films using laser ablation. A structural characterization using X-ray diffraction and an optical characterization using TRPL and TRMC demonstrated that the synthesized CsPbBr_3_ films exhibited a significantly high crystallinity. In particular, TRMC measurements demonstrated that the CsPbBr_3_ films had a high effective mobility of 2.47 cm^2^/Vs and long photocarrier lifetime of 16.5 μs (comparable to that of bulk single crystals). This indicates that the process suppresses the formation of defective structures that cause non-radiative recombination. This thin film process using laser ablation is also suitable for fabricating halide perovskite film heterostructures with organics and oxides. These have been demonstrated for the fabrication of LED devices. The fabrication of CsPbBr_3_ thin films sandwiched between an ETL of Mg_0.3_Zn_0.7_O and HTL of α-NPD caused the LED devices to emit green light. The film process using laser ablation enables the creation of thin films while controlling materials at the atomic level. The formation of new heterostructure interfaces is likely to result in the development of device materials with novel functionalities.

## Data Availability

The data that support the findings of this study are available from the corresponding authors upon reasonable request.
